# Mucin-2 knockout is a model of intercellular junction defects, mitochondrial damage and ATP depletion in the intestinal epithelium

**DOI:** 10.1038/s41598-020-78141-4

**Published:** 2020-12-03

**Authors:** Mariya A. Borisova, Kseniya M. Achasova, Ksenia N. Morozova, Evgeniya N. Andreyeva, Ekaterina A. Litvinova, Anna A. Ogienko, Maryana V. Morozova, Mariya B. Berkaeva, Elena Kiseleva, Elena N. Kozhevnikova

**Affiliations:** 1 The Federal Research Center Institute of Cytology and Genetics of the Siberian Branch of the Russian Academy of Sciences, Novosibirsk, Russian Federation 630090; 2 Institute of Molecular and Cellular Biology, The Siberian Branch of the Russian Academy of Sciences, Novosibirsk, Russian Federation 630090; 3 Scientific Research Institute of Physiology and Basic Medicine, Novosibirsk, Russian Federation 630117; 4 Siberian Federal Scientific Centre of Agro-BioTechnologies of the Russian Academy of Sciences, Krasnoobsk, Novosibirsk region, Russian Federation 630501

**Keywords:** Mechanisms of disease, Tight junctions, Gastrointestinal models

## Abstract

The disruption of the protective intestinal barrier—the ‘leaky gut’—is a common complication of the inflammatory bowel disease. There is limited data on the mechanisms of the intestinal barrier disruption upon low-grade inflammation characteristic of patients with inflammatory bowel disease in clinical remission. Thus, animal models that recapitulate the complexity of chronic intestinal inflammation in vivo are of particular interest. In this study, we used *Mucin-2* (*Muc2*) knockout mice predisposed to colitis to study intestinal barrier upon chronic inflammation. We used 4-kDa FITC-Dextran assay and transmission electron microscopy to demonstrate the increased intestinal permeability and morphological defects in intercellular junctions in *Muc2* knockout mice. Confocal microscopy revealed the disruption of the apical F-actin cytoskeleton and delocalization of tight junction protein Claudin-3 from the membrane. We further demonstrate mitochondrial damage, impaired oxygen consumption and the reduction of the intestinal ATP content in *Muc2* knockout mice. Finally, we show that chemically induced mitochondrial uncoupling in the wild type mice mimics the intestinal barrier disruption in vivo and causes partial loss of F-actin and membrane localization of Claudin-3. We propose that mitochondrial damage and metabolic shifts during chronic inflammation contribute to the leaky gut syndrome in *Muc2* knockout animal model of colitis.

## Introduction

One of the prominent features of the inflammatory bowel disease (IBD), including Crohn’s disease (CD) and ulcerative colitis (UC) is the disruption of the intestinal protective barrier known as the leaky gut syndrome^1^. Numerous reports demonstrate that leaky gut is associated with the morphological defects in the intercellular contacts of the epithelial cells of UC and CD patients^1–4^. These contacts comprise tight junctions (TJs), adherens junctions (AJs) and desmosomes^5^. AJs and desmosomes predominantly provide physical attachments between epithelial cells. TJs control size- and charge-selective paracellular transport and passive gradient-directed flow of some solutes and water^6,7^. TJs are formed in the apical part of the intestinal epithelium cells and consist of transmembrane proteins that enable an impermeable intercellular connection and junction adhesion molecules that connect TJs to the intracellular actomyosin network^8,9^. Filamentous actin (F-actin) connected to myosin forms the cytoskeletal basis for the TJs and AJs complexes^10^. Actin polymerization is regulated by the small Rho GTPases in response to the cell signaling events via phosphorylation/dephosphorylation by ROCK kinases and coordinated action of ADF/cofilin, profilin, Arp2/3, and other adapter molecules^11,12^. In turn, actomyosin contractility and apical cytoskeleton dynamics controls the integrity of AJs and TJs^13–15^.

The increased intestinal permeability is induced during inflammation by the pro-inflammatory cytokines during the active phase of the disease^16–18^. Some pathogenic microorganisms are also able to disrupt the intestinal barrier via regulation of Rho signaling or by myosin phosphorylation^19–22^. At the same time, the leaky gut is still very common in chronic colitis and IBD that is not associated with pathogenic infection or acute inflammation^23–26^, which raises the question of the mechanisms underlying the intestinal barrier disruption during remission. Moreover, studies involving CD patients revealed genetically determined increased intestinal permeability in their healthy first-degree relatives, which could not be explained with cytokine- or pathogen-induced intestinal barrier disruption^27,28^.

Multiple reports show strong metabolic deregulation, mitochondrial damage and ATP depletion in chronic UC patients^29–35^. Structural defects have been described for mitochondria, cytoskeleton and cell-to-cell junctional complexes^2,3,31,36,37^ in the intestinal samples of patients as revealed by electron microscopy. In vitro studies demonstrate that ATP depletion itself causes immediate effects on the barrier integrity via F-actin disassembly and disintegration of the TJ protein complexes^38–41^. Thus, an alternative mechanism of the intestinal epithelial permeability during chronic inflammation might involve mitochondrial damage and metabolic deficiency^40,42,43^. However, the validity of this hypothesis still requires experimental support using appropriate in vivo experimental models.

*Muc2* knockout mouse strain is one of the well-known experimental models of chronic colitis and colon cancer^44,45^. *Muc2*^*−/−*^ mice lack Mucin2^44^—the major secreted intestinal mucin that prevents intestinal bacteria from translocating into the intestinal tissues^46^. Due to Mucin-2 deficiency, intestinal epithelial cells remain in direct contact with bacteria, which leads to the immune system activation and inflammation^47^. *Muc2* knockout mouse model was demonstrated to well recapitulate the inflammatory state of the intestine found in IBD patients with low-grade inflammation in terms of the immune response and intestinal morphology^48^. However, the data regarding the leaky gut phenotype in *Muc2*^*−/−*^ mice remains contradictory: several studies reported no intestinal barrier disruption^49–51^, and one paper showed a modest increase in the gut permeability upon *Muc2* mutation^52^. Only experimental infection with pathogenic microorganisms like *Citrobacter rodentium* and *Salmonella enterica* was clearly shown to induce acute inflammation and the intestinal barrier permeability in *Muc2*^*−/−*^ mice^49,51^. Thus, it is important to use an alternative and unbiased approach to study the state of the intestinal protective barrier in *Muc2* knockout model.

Here we combined transmission electron microscopy (TEM) with the in vivo intestinal permeability assay to demonstrate that *Muc2*^*−/−*^ mice in the state of chronic inflammation develop intestinal barrier defects. Our data shows that these abnormalities are associated with the structural impairment of the TJs in *Muc2*^*−/−*^ mice. These defects are accompanied by the disruption of the apical F-actin cytoskeleton and delocalization of a TJ component Claudin-3 from the membrane. We further demonstrate mitochondrial damage in the intestinal epithelium associated with the reduction of ATP content upon *Muc2* knockout. Chemically induced mitochondrial uncoupling in the otherwise untreated C57Bl/6 mice induced the intestinal barrier disruption in vivo and caused loss of F-actin and Claudin-3 disassembly similar to that found in *Muc2*^*−/−*^ mice. Given these results, we propose that mitochondrial damage and metabolic defects during chronic inflammation promote the leaky gut syndrome in *Muc2* knockout animal model of colitis.

## Results

### Mucin 2 knockout is a mouse model of chronic intestinal inflammation and leaky gut

**Figure 1:**
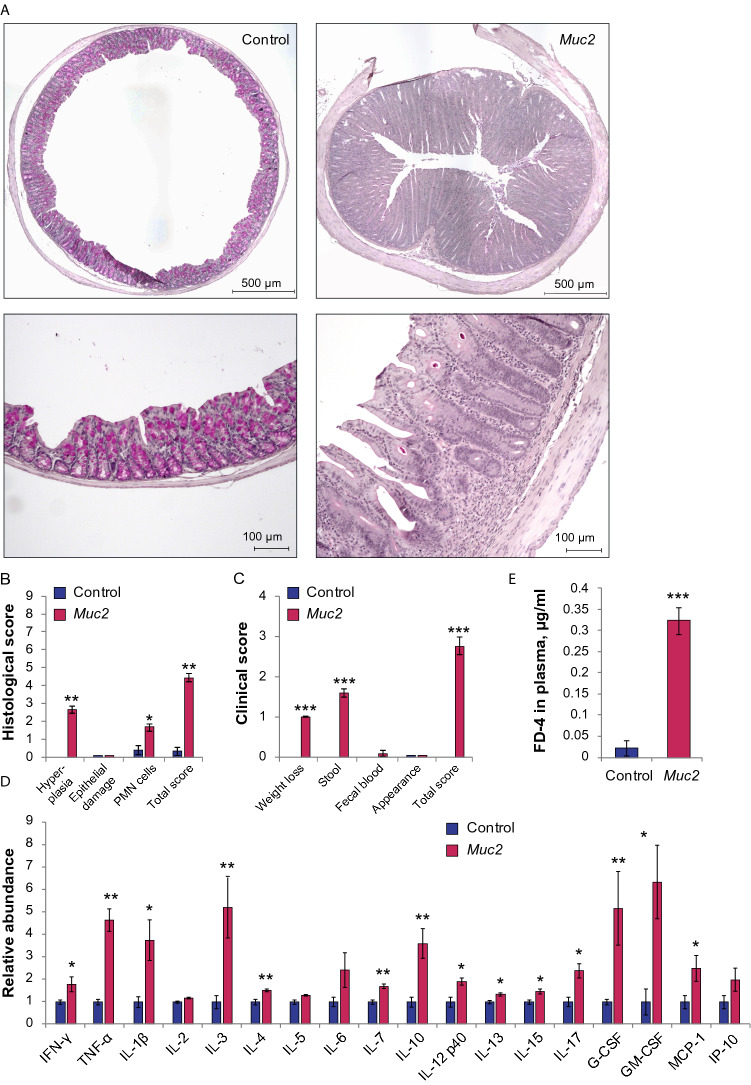
*Muc2* knockout as a model of chronic intestinal inflammation and leaky gut. (**A**) PAS-stained colonic sections of C57Bl/6 and *Muc2*^*−/−*^ mice. (**B**) Histological scoring of the inflammatory response. *p < 0.05, **p < 0.01, *vs* C57Bl/6, Mann–Whitney *u*-test. (**C**) Clinical scoring of the inflammatory response. *p < 0.05, **p < 0.01, *vs* C57Bl/6, Mann–Whitney *u*-test. (**D**) Cytokine levels in the descending colon. *p < 0.05, ** = *p* < 0.01, ***p < 0.001, *vs* C57Bl/6, Mann–Whitney *u*-test. (**E**) Intestinal permeability. ***p < 0.001, *vs* C57Bl/6, Student’s t-test.

We first aimed to investigate whether the state of intestinal inflammation in the descending colon of adult, specific pathogen free (SPF) *Muc2*^*−/−*^ mice in our housing conditions is comparable to that found previously^44,45^. Histological examination revealed profound chronic intestinal inflammation in the descending colon of the *Muc2*^*−/−*^ mice in comparison to C57Bl/6 control mice (Fig. 1A). Goblet cells were depleted of the mucus content indicating the loss-off-function mutation in the *Muc2* gene. Colonic crypts were significantly longer in *Muc2*^*−/−*^ mice due to the over-proliferation of epithelial cells (for both middle third and upper third parts of the crypt: Z = 1.96, p = 0.0497, Mann–Whitney *u* test, Fig. S1), as described previously^44,45^, so that hyperplasia score was higher in *Muc2*^*−/−*^ mice (Z = 2.80, *p* = 0.005, Mann–Whitney *U* test, Fig. 1B). Polymorphonuclear (PMN) cells infiltration was significantly higher in *Muc2*^*−/−*^ mice in comparison to C57Bl/6 indicating a strong inflammatory immune reaction (Z = 2.28, *p* = 0.022, Mann–Whitney *u* test, Fig. 1B). However, there were no obvious edemas or ulcerations, and the overall integrity of colonic epithelium was normal. The total inflammatory score was higher in *Muc2* knockout than in C57Bl/6 mice (Z = 2.80, *p* = 0.005, Mann–Whitney *u* test, Fig. 1B). These data indicate that the state of inflammation we observed in uninfected *Muc2*^*−/−*^ mice resembled modest inflammation without apparent tissue damage.

This observation was further supported by the clinical score, which showed significant changes in the physiological state of the mutant animals. The overall appearance and activity of the mutant mice were normal. However, *Muc2*^*−/−*^ knockout animals lost weight (Z = 3.31, *p* < 0.001, Mann–Whitney *u* test, Fig. 1C) and suffered from diarrhea (Z = 3.31, *p* < 0.001, Mann–Whitney *u* test, Fig. 1C), but without prominent blood in the stool. The overall clinical score was significantly higher in the mutants as compared to C57Bl/6 (Z = 3.31, *p* < 0.001, Mann–Whitney *u* test, Fig. 1C), suggesting clinical relevance of this mouse model.

We further characterized the inflammatory state of the intestine upon *Muc2* knockout using cytokine profiling. We found that many of the cytokines were up-regulated in the mutant mice as compared to the control suggesting the overall activation of the mucosal immune system. For instance, pro-inflammatory cytokines IFN-γ, Il-1β, TNF-α, Il-17, and MCP-1 were significantly up-regulated in *Muc2*^*−/−*^ mice (IFN-γ: Z = 2.10, *p* = 0.036; Il-1β: Z = 2.46, *p* = 0.014; TNF-α: Z = 2.65, *p* = 0.008; Il-17: Z = 2.46, *p* = 0.014; MCP-1: Z = 2.10, p = 0.036; Mann–Whitney *u* test, Fig. 1D). However, the anti-inflammatory cytokines or the ones associated with both: pro- and anti-inflammatory immune responses were also up-regulated (Il-3: Z = 2.65, *p* = 0.008; Il-4: Z = 2.65, *p* = 0.008; Il-10: Z = 2.65, *p* = 0.008; Il-13: Z = 2.28, *p* = 0.22; Mann–Whitney *u* test, Fig. 1D). G-CSF and GM-CSF cytokines involved in cell proliferation were significantly elevated upon *Muc2* knockout (G-CSF: Z = 2.65, *p* = 0.008; GM-CSF: Z = 2.28, *p* = 0.022; Mann–Whitney *u* test, Fig. 1D). We also observed a significant increase in Il-7, Il-12p40, and Il-15 (Il-7: Z = 2.65, *p* = 0.008; Il-12p40: Z = 2.28, *p* = 0.022; Il-15: Z = 2.46, *p* = 0.014; Mann–Whitney *u* test, Fig. 1D). These data demonstrate that both: pro- and anti-inflammatory immune responses were activated in the intestine of *Muc2*^*−/−*^ mice. We then tested if another feature of low-grade inflammation—an increased intestinal barrier permeability—was characteristic of this mouse model of colitis. Thus, we investigated if the *Muc2*^*−/−*^ intestine was permeable for a 4-kDa FITC-Dextran molecule, which should not normally penetrate the intestinal barrier. After the oral gavage, we found that 4-kDa FITC-Dextran was detected in the blood plasma of *Muc2*^*−/−*^, but not of the control mice (t = 7.65, *p* < 0.001, Student’s t-test, Fig. 1E). Additionally, intestinal permeability for 4-kDa FITC-Dextran was also detected in 10 week- (Z = 2.55, p = 0.011, Mann–Whitney *u*-test, Fig. S1) and in 18–20 week-old *Muc2*^*−/−*^ mice (Z = 2.33, p = 0.02, Mann–Whitney *u* test, Fig. S1). In order to specifically test the colonic permeability, we performed rectal administration of 4-kDa FITC-Dextran and found a significant barrier dysfunction in *Muc2* mutant mice as compared to the control (Z = 2.55, p = 0.013, Mann–Whitney *u*-test, Fig. S1). Taken together, these experiments suggest that SPF *Muc2*^*−/−*^ mice represent an accurate model of inflammation.

We have further questioned whether the intestinal barrier dysfunction is attributed to the *Muc2* knockout itself or was influenced by the associated shift in the microflora composition. Thus, we investigated barrier integrity by 4-kDa FITC-Dextran oral gavage in the *Muc2*^*−/−*^ wild-type littermates (*Muc2*^+*/*+^), and found that their intestinal permeability was undistinguishable from that of C57Bl/6 mice (Fig. S1). This result demonstrates that possible deviations in microflora within *Muc2* breeding colony are unlikely to cause intestinal barrier damage.

### Increased intestinal permeability results from the loss of structural integrity in intercelular junctions

**Figure 2:**
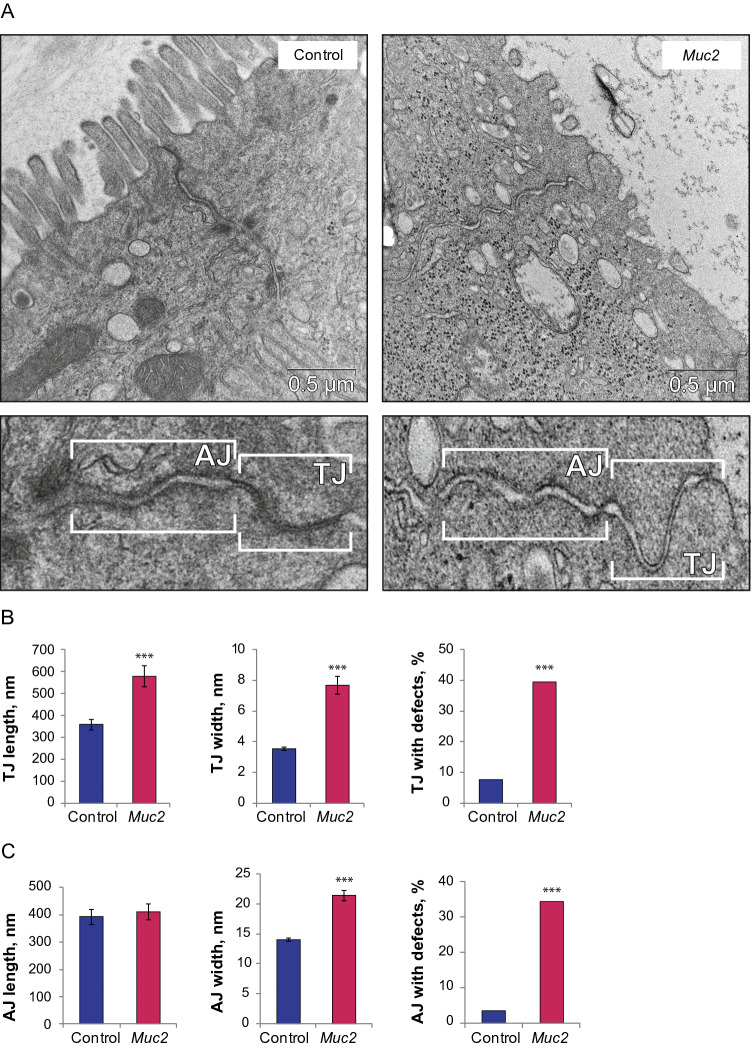
Increased intestinal permeability results from lack of structural integrity in TJs. (**A**) TEM of the descending colon epithelium of C57Bl/6 and *Muc2*^*−/−*^ mice. (**B**) TJ length (***p < 0.001, Mann–Whitney *u* test), width (***p < 0.001, Mann–Whitney *u* test) and the percentage of the defective TJs (***p < 0.001, *χ*^2^ test). (**C**) AJ length, width (***p < 0.001, Student’s t-test) and the percentage of the defective AJs (***p < 0.001, *χ*^2^ test).

Given that the intestinal permeability data in the previous studies on Muc2^−/−^ mouse model was contradictory^49–52^, we assessed morphological changes in the intestinal epithelium using TEM. There was no apparent tissue damage or gaps in the epithelial cell array, so that the overall epithelial integrity appeared normal. Fine analysis of the ultrastructures revealed that TJ and AJ morphology was altered in the knockout mice (Fig. 2A). While in the control mice TJs appeared as tightly sealed apical membranes of the neighboring cells, in *Muc2*^*−/−*^ mice, TJs were loose, with wide gaps between the membranes of the adjacent cells (Fig. 2A, S2). In some cases, the membranes of the neighboring cells were fully open in the apical part, or bubble-like openings were found within TJs (Fig. S2). AJs also appeared wider with less density in the intercellular spaces (Fig. 2A). Morphometric analysis confirmed our observations: the width of the TJs and AJs was significantly wider in the colon of *Muc2*^*−/−*^ animals in comparison to the control (TJs: Z = 7.25, *p* < 0.001, Mann–Whitney *u* test; AJs: t = 6.27, *p* < 0.001, Student’s t-test, Fig. 2B,C). The percentage of TJs and AJs with defects was also higher in *Muc2*^*−/−*^ mice as compared to the control (TJ: p < 0.001, *χ*^2^ test; AJ: *p* < 0.001, *χ*^2^ test, Fig. 2B,C). At the same time, TJs in *Muc2*^*−/−*^ mice were significantly longer (Z = 3.62, *p* < 0.001, Mann–Whitney *u* test, Fig. 2B) than in the control mice. We have also noticed that *Muc2* knockout affected desmosome number and morphology (Fig. S3). The number of desmosomes per lateral cell membrane was significantly higher in the mutant mice (t = 3.82, *p* < 0.001, Student’s t-test, Fig. S3), and the percentage of defective desmosomes was increased (*p* < 0.001, *χ*^2^ test, Fig S3). Desmosome defects were mostly seen as semi-desmosomes or incompletely formed desmosomes (Fig. S3). Moreover, we found that the intercellular spaces in the intestinal epithelium were significantly wider in *Muc2*^*−/−*^ mice as compared to the control (t = 7.72, *p* < 0.001, Student’s t-test, Fig. S3) with frequent broad disjunctions. While on average the width of an intercellular space in the control was about 15 nm, more than 25% of the intercellular spaces in *Muc2*^*−/−*^ mice were wider than 25 nm (*p* < 0.001, *χ*^2^ test, Fig. S3). Since TJs control size- and charge-selective paracellular transport, the increased intestinal permeability in *Muc2*^*−/−*^ mice can be attributed to the structural defects in TJs. However, the other defects described above suggest the overall loss of cell-to-cell contact in the epithelium of the knockout mice.

As pro-inflammatory cytokines were up-regulated in *Muc2*^*−/−*^ mice, we performed gene expression analyses of the genes involved in the intestinal barrier formation that are known to change expression under the inflammatory conditions. However, we found that neither TJ genes nor the AJ genes changed expression in *Muc2*^*−/−*^ mice in comparison to the control (Fig. S1). Gene expression of matrix metalloproteinases *Mmp2* and *Mmp9*, which may contribute to the increased intestinal permeability, was not altered upon *Muc2* knockout either (Fig. S1). Furthermore, neither *leukotriene C4 synthase* (*Ltc4*) gene expression nor β-catenin downstream target genes *mitochondrially encoded NADH dehydrogenase 2* (*Nd2*) and *mitochondrially encoded NADH dehydrogenase 6* (*Nd6*) significantly changed upon *Muc2* knockout (Fig. S1). These results demonstrate that the intestinal barrier dysfunction in *Muc2*^*−/−*^ mice is unlikely to be attributed to transcriptional regulation by the pro-inflammatory cytokines.

### Brush border defects, mitochondrial damage and reduced ATP content are characteristic of the Muc2 knockout model of colitis

**Figure 3:**
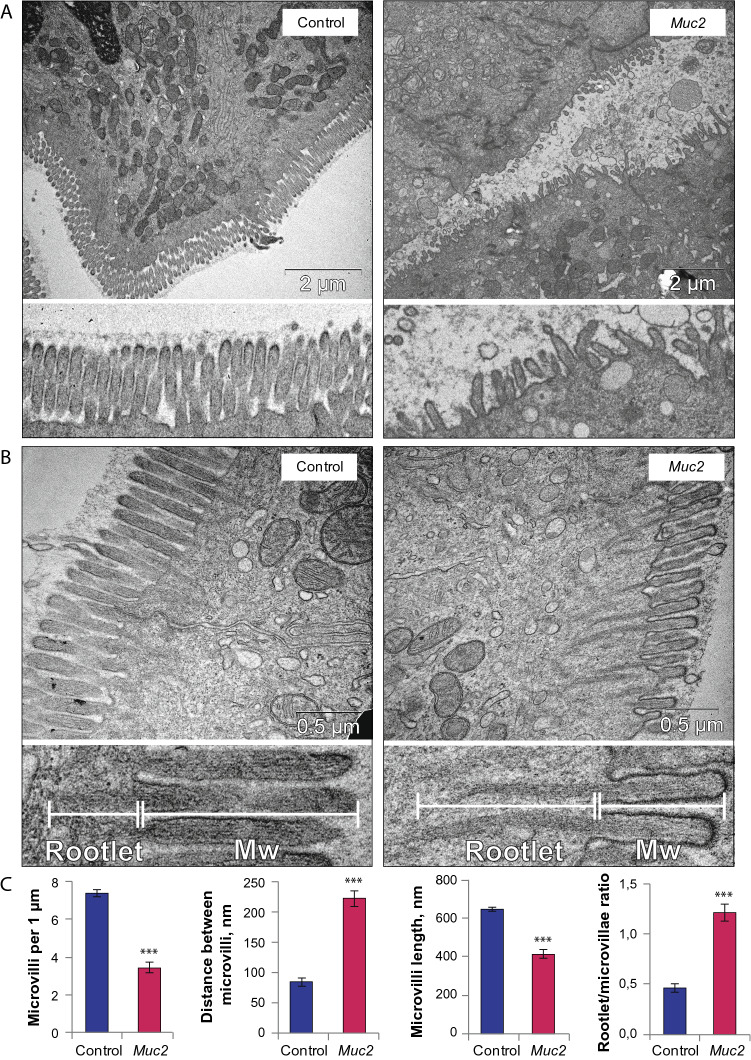
Defective microvilli upon *Muc2* knockout. (**A**) TEM of the microvilli in the descending colon epithelium of C57Bl/6 and *Muc2*^*−/−*^ mice. (**B**) The detailed view of the microvilli structure. (**C**) Morphometric analysis: microvilli number per 1 µm of cell surface (***p < 0.001, Mann–Whitney *u* test), distance between microvilli (***p < 0.001, Mann–Whitney *u* test), microvillus length (***p < 0.001, Student’s t-test), rootlet/microvillus length ratio (***p < 0.001, Student’s t-test). *Mw* microvillus.

Along with the defects in TJs and AJs, we have also found other morphological abnormalities in the intestinal epithelial cells. In particular, *Muc2* knockout resulted in a strong depletion of microvillus brush border from the apical cell surface, so that in some regions of the intestine there were no structurally defined microvilli (Figs. 2A, 3). In contrast, in the control animals the microvilli formed a very regular and well-organized array. Morphometric analysis confirmed that there were less microvilli per 1 µm of cell surface in the knockout mice (Z = 6.30, p < 0.001, Mann–Whitney *u* test, Fig. 3C), and the average distance between the neighboring microvilli was larger in the knockout mice as compared to the control (Z = 7.27, *p* < 0.001, Mann–Whitney *u* test, Fig. 3C). Moreover, in the regions where microvilli were observed in *Muc2*^*−/−*^ mice, their morphology was substantially different from those in the control (Fig. 3B). Microvilli were generally shorter in *Muc2*^*−/−*^ mice (t = 9.30, *p* < 0.001, Student’s t-test, Fig. 3C). Actin filaments that form a microvillus scaffold were embedded deeply into the cell body, so that the rootlet of the microvillus was longer than the microvillus itself, and the rootlet to microvillus length ratio was higher in the knockout mice (t = 8.02, *p* < 0.001, Student’s t-test, Fig. 3B,C).

**Figure 4:**
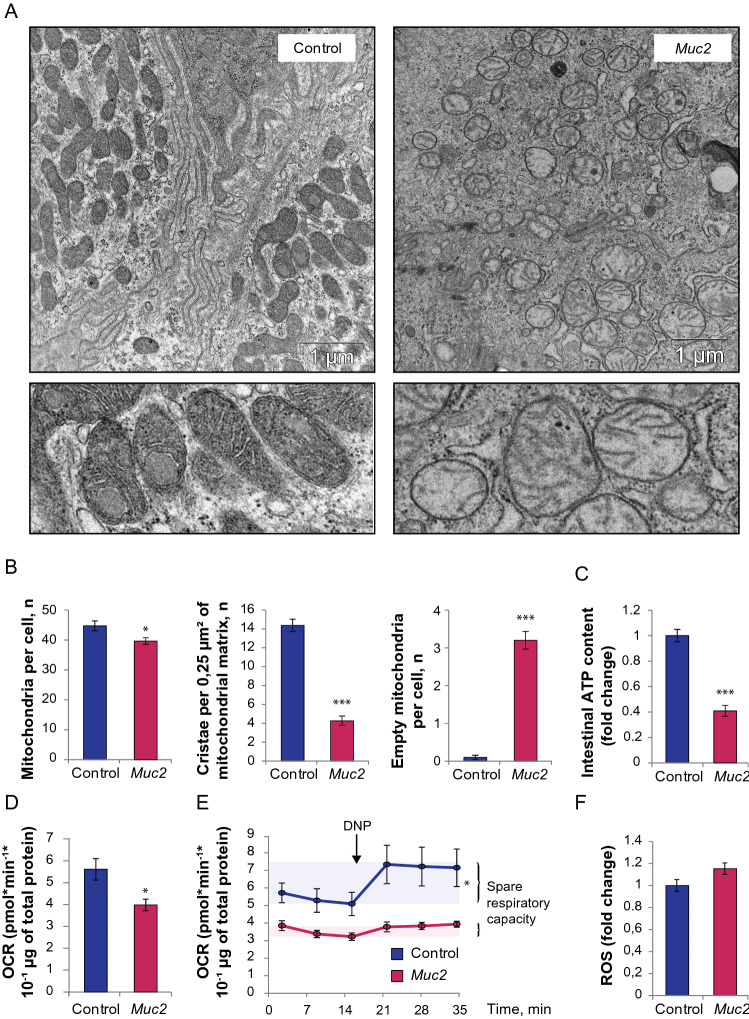
Mitochondrial damage and the reduction of ATP content and OCR in the descending colon in *Muc2* knockout. (**A**) TEM of the mitochondria in the descending colon epithelium of C57Bl/6 and *Muc2*^*−/−*^ mice. (**B**) Morphometric analysis: number of mitochondria per cell (*p < 0.05, *vs* C57Bl/6, Student’s t-test), number of cristae per 0.25 μm^2^ of mitochondrial matrix, ***p < 0.001, *vs* C57Bl/6, Student’s t-test), number of empty mitochondria per cell (***p < 0.001, *vs* C57Bl/6, Mann–Whitney *u*-test). (**C**) ATP level in the descending colon. ***p < 0.001, *vs* C57Bl/6, Student’s t-test. (**D**) Baseline OCR in the mitochondria of the isolated colonic crypts (*p < 0.05, *vs* C57Bl/6, Mann–Whitney *u*-test). (**E**) Spare respiratory capacity determined upon DNP treatment of the isolated colonic crypts (*p < 0.05 for C57Bl/6, Friedman test). (**F**) ROS formation in the isolated colonic crypts.

Another prominent feature of the *Muc2*^*−/−*^ epithelium revealed by TEM was a drastic change in the morphology of mitochondria (Fig. 4A). Generally, they were round in shape and depleted of cristae and electron-dense content as compared to the control. We found fewer mitochondria per cell in the intestinal epithelium of *Muc2*^*−/−*^ mice (t = 2.23, *p* = 0.03, Student’s t-test, Fig. 4B). Mutant mitochondria contained less cristae in comparison to the control (t = 10.28, *p* < 0.001, Student’s t-test, Fig. 4B), and empty mitochondria were detected more often in *Muc2*^*−/−*^ mice (Z = 6.02, *p* < 0.001, Mann–Whitney *u* test, Fig. 4B). Mitochondrial damage was accompanied by a substantial reduction in ATP content in the descending colon (t = 8.88, *p* < 0.001, Student’s t-test, Fig. 4C) indicative of mitochondrial dysfunction upon *Muc2* knockout.

We utilized oxygen consumption rate (OCR) analysis in isolated colonic crypts to evaluate mitochondrial metabolism by Seahorse technology (Agilent). We found that OCR was significantly lower in *Muc2*-derived crypts as compared to the control (Z = 1.96, *p* = 0.049, Mann–Whitney *u* test, Fig. 4D). 2,4-dinitrophenol **(**DNP) was used to induce proton leak, which uncouples ATP synthase from the proton pump, and measure spare respiratory capacity of the mitochondria. We found that *Muc2*-derived crypts were unable to increase OCR upon DNP treatment, which indicates severe defects in mitochondrial metabolism. At the same time, control crypts increased OCR in response to DNP as expected (χ^2^(5) = 12.9, *p* = 0.024, Friedman test, Fig. 4E).

As mitochondrial damage can arise from reactive oxygen species (ROS) production in the inflammatory environment, we stained the freshly isolated colonic crypts with a ROS-sensitive CellROX dye. Fluorescence intensity analysis showed that there was no significant change in ROS production during chronic inflammation caused by the *Muc2* mutation (Fig. 4F).

### Structural defects in intercellular junctions are accompanied by the lack of F-actin polymerisation at the epithelial cell surface

**Figure 5:**
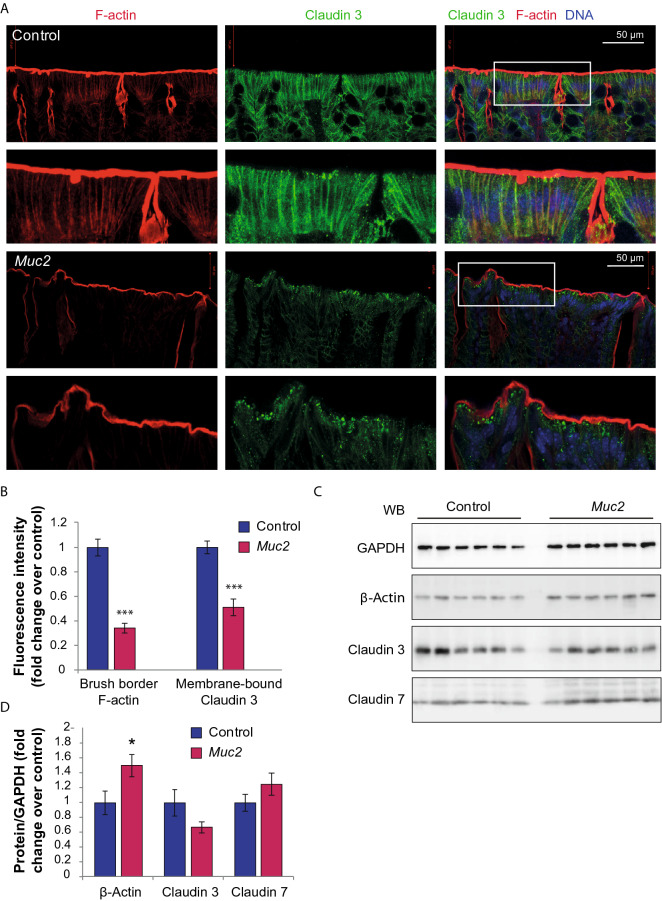
Lack of F-actin polymerization at the epithelial cell surface and Claudin-3 delocalization upon *Muc2* knockout. (**A**) Claudin-3 and F-actin double immunostaining in the descending colon of C57Bl/6 and *Muc2*^*−/−*^ mice. Maximum intensity projections through 40 µm of tissue are shown for each image. (**B**) Fluorescence intensity quantification of Claudin 3 along the cell membrane and F-actin within the brush border (***p < 0.001, *vs* C57Bl/6, Student’s t-test). (**C**) Western blot analysis of the total protein in colonic samples. (D) Quantification of the Western blot data (normalized to GAPDH protein level, fold change), *p < 0.05, *vs* C57Bl/6, Mann–Whitney *u*-test.

The described above morphological abnormalities suggest that the structural basis of the microvilli—F-actin filamentous bundles—were disrupted upon *Muc2* knockout. In order to test this hypothesis, we employed confocal microscopy to evaluate the F-actin content in the apical membrane of the epithelial cells using fluorescently labelled phalloidin staining that specifically binds to F-actin. As expected, we found a very strong phalloidin binding along the microvillus brush border in the apical surface of the intestinal cells in the control (Fig. 5A). However, we observed a lot weaker and diffuse phalloidin signal in the epithelial surfaces of the *Muc2*^*−/−*^ mice, (t = 7.39, *p* < 0.001, Student’s t-test, Fig. 5A,B). We have also used TJ subunit Claudin-3 immunostaining as a marker of TJ protein complexes to identify its subcellular localization. We found that in the control, Claudin-3 was mostly bound to the lateral membranes of the epithelial cells (Fig. 5A). In contrast, there was significantly less membrane-bound Claudin-3 in the knockout mice (t = 5.75, *p* < 0.001, Student’s t-test, Fig. 5B) and most of the Claudin-3 protein content was found in the cytoplasm. Moreover, DNA staining revealed that in some regions of the intestine, epithelial cells in the *Muc2*^*−/−*^ mutants lost structural arrangement along the crypt surface characteristic to polarized epithelium as was seen in the control (Fig. 5A). β-actin level was slightly increased in *Muc2*^*−/−*^ mice in comparison to C57Bl/6 (Z = 2.32, p = 0.020, Mann–Whitney *u* test, Fig. 5D), as shown by Western-blot analysis, whereas Claudin-3 showed no change (Fig. 5D). In agreement with the gene expression data, Claudin 7 protein content in the colon was unchanged (Fig. 5D), supporting our hypothesis that down-regulation of TJ gene expression is unlikely to cause the disruption of the intestinal barrier. These data suggest that TJ defects may arise from the impaired dynamics of F-actin in the apical surface of the cells leading to the increased intestinal permeability, disrupted cell-to-cell communication and loss of epithelial structure.

### Chemically induced mitochondrial uncoupling in the wild type mice induced the intestinal barrier disruption in vivo and caused partial loss of F-actin

**Figure 6:**
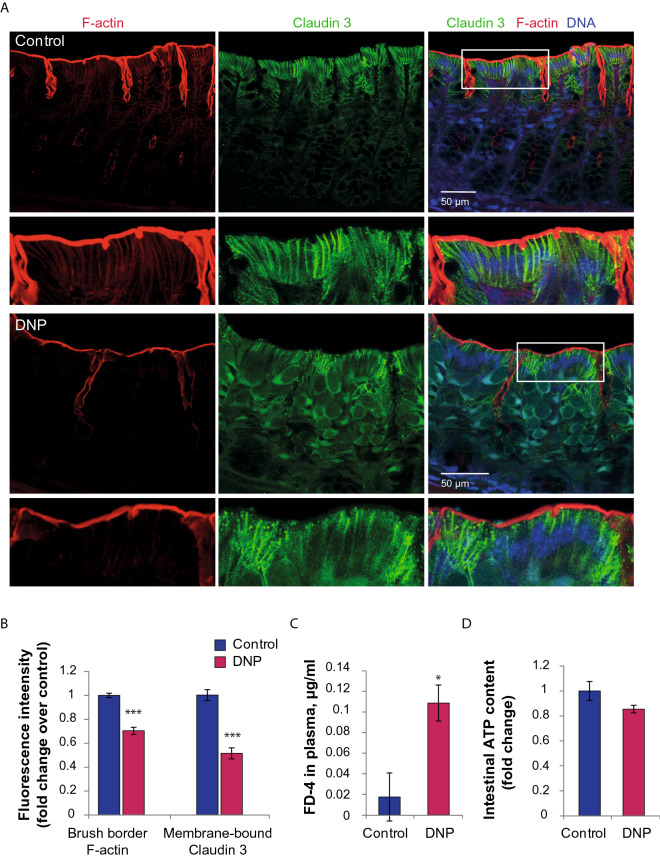
Chemically induced mitochondrial uncoupling in the C57Bl/6 mice induced the intestinal barrier disruption in vivo and decreased F-actin polymerization. (**A**) Claudin-3 and F-actin double immunostaining in the descending colon of C57Bl/6 mice, and in C57Bl/6 mice treated with 0.8 g/l DNP. Maximum intensity projections through 40 µm of tissue are shown for each image. (**B**) Fluorescence intensity quantification of Claudin 3 along the cell membrane and F-actin within the brush border (***p < 0.001, *vs* C57Bl/6, Student’s t-test). (**C**) Intestinal permeability (*p < 0.05, *vs* C57Bl/6, Student’s t-test). (**D**) ATP levels in the descending colon.

Since mitochondrial dysfunction and ATP depletion are known to affect F-actin, TJ and intestinal permeability, we used DNP as a mitochondrial uncoupler to induce mitochondrial damage and metabolic stress in the otherwise intact C57Bl/6 mice. We found a strong Claudin-3 straining in the cytoplasm upon DNP treatment, whereas it was almost exclusively membrane-bound in the control. At the same time, many cells in the epithelium of DNP-treated mice had little membrane-bound Claudin-3 in comparison to the control with a distinct membrane staining for Claudin-3 (t = 7.42, *p* < 0.001, Student’s t-test, Fig. 6A,B). Phalloidin signal in the microvillus brush border of DNP-treated mice was less intence as compared to the control indicative for the reduction in the F-actin content (t = 8.28, *p* < 0.001, Student’s t-test, Fig. 6A,B). DNP treatment resulted in the increased intestinal permeability to 4 kDa FITC-Dextran in comparison to C57Bl/6 (t = 2.80, p = 0.019, Student’s t-test, Fig. 6C). Intestinal ATP content was generally lower upon DNP treatment, although, no statistical significance was detected (Fig. 6D).

## Discussion

One of the widely used mouse models of IBD is *Muc2* knockout mice that tend to develop spontaneous colitis followed by the formation of adenocarcinomas^44,45,53^. In the present study, we used this mouse model to evaluate the intestinal barrier permeability upon chronic colitis. Since mouse strain, animal housing and health status significantly affect the severity of colitis^54,55^, we first investigated whether *Muc2*^*−/−*^ mice in the present study exhibited intestinal inflammation as was described previously^44,45,47,53,56^. Histological analysis together with the intestinal cytokine profiling revealed that, indeed, mutant mice develop modest chronic inflammation. Histological examination revealed crypt hyperplasia due to the elevated proliferative activity in the epithelium (Fig. S1) ^44^, and a significant PMN cells infiltration (Fig. 1A,B), but no apparent epithelial damage. Clinical score was significantly higher in the mutant mice (Fig. 1C). We also observed a significant up-regulation of the pro-inflammatory cytokines TNF-a, IL-1β, IL-12p40, and IL-17, and an anti-inflammatory cytokine IL-10 (Fig. 1D). Up-regulation of IL-3 might also indicate the anti-inflammatory response^57^. Most probably, this cytokine together with IL-10 favor the low-grade inflammation in the absence of MUC2. These data agree with the previous studies involving *Muc2* mutants^45,53^ and resemble the features of inflammation in IBD patients^58–61^. Human studies suggest that the intestinal barrier disruption is a prominent feature of chronic inflammation^23,24,62,63^. However, the data on *Muc2* model regarding the leaky gut phenotype was contradictory: independent researchers reported no effect on the intestinal barrier integrity^49–51^, whereas one study reported a modest increase in the gut permeability upon *Muc2* mutation^52^. Substantial disruption of the intestinal barrier in *Muc2*^*−/−*^ mice was reported only upon experimental infection with *Citrobacter rodentium*^49^, *Salmonella enterica*^51^ or *Entamoeba histolytica*^50^. The discrepancy between the previous reports might be due to the health status of the animals or different normalization approaches in the permeability assay that were not explained in detail in these studies. In the present work, we observed a significant intestinal permeability to 4-kDa FITC-Dextran in SPF *Muc2*^*−/−*^ male mice (Fig. 1E), which was accompanied by the structural defects in TJs as revealed by TEM (Fig. 2). A number of structural abnormalities were seen in the TJs of the mutant mice including full opening, ‘bubbling’, and increased width of the TJs (Fig. S2). Similar defects were found in previous studies upon loss-of-function mutations in TJ components^64,65^, bacterial infection^66,67^, inflammation, IBD and CD^68–70^. These structural impairment lead to the increased intestinal permeability and explain the leaky gut phenotype upon *Muc2* mutation. Together, these data allow concluding that, indeed, *Muc2*^*−/−*^ mouse strain recapitulates the leaky gut phenotype upon modest inflammation and agrees with clinical data on IBD patients.

Morphological analysis of the cellular ultrastructures by TEM revealed other characteristic features that were previously found in patients with IBD^36,37^. Interestingly, we found that microvilli were drastically disrupted in *Muc2*^*−/−*^ mutants: they were shorter in length, irregular and partially absent from the epithelial surface (Fig. 3). Moreover, we found that filamentous scaffolds of the microvilli ‘sunk’ into the cytoplasm so that the rootlet of the microvillus was longer than its length (Fig. 3), whereas in the control, the rootlet was about a half of the microvillus length. This observation led us to the conclusion that the structure of the cytoskeletal components responsible for microvilli formation is disturbed. F-actin bundles comprise the structural basis of the microvillus^71,72^, so the defects in F-actin dynamics might be involved in microvilli degeneration found upon *Muc2* knockout. For instance, similar brush border defects are found upon knockdown of proteins involved in F-actin network assembly^73^ and in patients with IBD^37^. Moreover, AJ disruption (Fig. 2) and intercellular space enlargement (Fig. S3), though not directly involved in the leaky gut phenotype, are suggestive of the loss in cytoskeleton integrity. Our confocal microscopy data supported this hypothesis since there was a strong reduction of F-actin in the intestinal epithelial cell surface (Fig. 5) in *Muc2*^*−/−*^ mice. These data agree with the previous findings that pharmacological or genetic disruption of the actin cytoskeleton results in AJ/TJ disassembly and the disruption of epithelial barriers^74–76^. We did not find any reduction of total actin in the intestine of the mutant animals as can be seen from the Western Blot analysis (Fig. 5), and proposed that actin dynamics might be affected in *Muc2* mice. Our data demonstrates that Claudin-3 used as a TJ marker was dissociated from the membrane and redistributed into the cytoplasm in *Muc2* mutant animals (Fig. 5) indicating the structural impairment of TJ complexes and supports with our findings in TEM. As was shown previously, chemically induced actin depolymerization leads to the TJ proteins redistribution and internalization by endocytosis, which coincides with a fall of transepithelial electrical resistance^75^. It is very likely that similar events occur in vivo, however, further experiments including TJ immunoprecipitation from the appropriate cellular fractions followed by proteomic analysis are needed to understand the protein dynamics of the actomyosin and TJs in *Muc2* knockout model of colitis.

Another prominent morphological feature of *Muc2* knockout mice is the drastic change in mitochondrial appearance: mitochondria were round in shape, lacked matrix density and had significantly lower number of cristae, with some of them having no cristae at all (Fig. 4). These structural defects were accompanied by the functional impairment of mitochondrial metabolism as revealed by the significantly lower ATP content in the intestine of *Muc2*^*−/−*^ mice (Fig. 4C). We also found that *Muc2* mutation leads to the reduction in OCR (Fig. 4D), which might originate from the loss of mitochondrial cristae and, as a result, lack of functional respiratory complexes. Moreover, the impaired mitochondrial oxidation in mutant epithelial cells seem to be the maximum of their capacity as the proton leak induced by DNP did not induce any increase in OCR. This indicates either severe damage of the respiratory complexes or a strong metabolic dysfunction in the overall mitochondrial biochemistry upon chronic inflammation and loss of mucus content. The metabolic defects including low ATP level as well as mitochondria impairment and dysfunction are very common in subjects with IBD^29,36^. Thus, apart from the chronic inflammation and intestinal permeability, *Muc2* knockout model recapitulates metabolic defects characteristic to human patients. Interestingly, mitochondrial damage or impairment of ATP production in cultured cells using mitochondrial uncoupling agents strongly affect epithelial barrier integrity, causing F-actin depolymerization, microvillus retraction and TJ protein components dissociation from the cellular membrane, forming insoluble protein aggregates^39–41,77^. Moreover, the uncoupling agent DNP was shown to alter intestinal mitochondrial morphology, increase intestinal permeability and cause inflammation in rats in vivo^78^. It was proposed that low ATP content downregulates phosphorylation of actin depolymerizing protein cofilin, resulting in its activation and leading to the depolymerization of F-actin^79^. Alternatively, myosin II dissociation from actin during severe ATP depletion was shown to contribute to destabilization of the actin cytoskeleton^80^. Here we used mitochondrial uncoupler DNP in the otherwise intact C57Bl/6 mice in order to demonstrate in vivo that mitochondrial damage can induce intestinal barrier permeability and cause the defects of F-actin and Claudin-3 distribution similar to that found in *Muc2*^*−/−*^ model of intestinal inflammation (Fig. 6). However, in our experimental setup, the ATP decrease in the intestine at the time of the permeability assay was insignificant, which might indicate that compensatory restoration of ATP precedes the epithelial sealing, or that there are other aspects of mitochondrial damage that affect barrier function. Another known consequence of the mitochondrial uncoupling is a change in cellular redox state and ROS production that also have impact on Rho activation^81^, actin dynamics^82^ and barrier function. Our data suggest that there is no significant change in ROS production in the colonic epithelial cells of *Muc2*^*−/−*^ mice (Fig. 4F) at least in the state of chronic colitis. Most probably, at this stage, the pro-inflammatory cytokines were no longer able to induce ROS due to the severe mitochondrial damage and reduced oxygen consumption. However, at the onset of inflammation, high ROS could initiate mitochondrial defects. Thus, the exact effect of mitochondrial uncoupling on the apical actomyosin cytoskeleton and Rho-dependent barrier dysfunction in chronic inflammation are yet to be elucidated and might be key to the leaky gut phenotype.

It is still unclear why mitochondrial damage is so prominent in the intestinal pathologies, whether it is a cause or a consequence of the disease. There is strong evidence that intestinal microbiota affects host metabolism^83^, and that microbial metabolic products like butyrate, which serves the main source of epithelial ATP by mitochondrial beta-oxidation, might contribute to the metabolic state of the intestine^84^. Moreover, exogenous butyrate has an ameliorating effect on intestinal permeability^85^. At the same time, microbial composition in *Muc2* mice is different from that in their wild type counterparts^86^ due to the lack of mucous environment. It is possible, that the supply of butyrate and other short chain fatty acids are limited in *Muc2*^*−/−*^ mice, which leads to the mitochondrial dysfunction in epithelial cells. As we found here, the wild-type littermates of *Muc2* mutant mice have no intestinal barrier defects (Fig. S1). This indicates that only microbiome shifts directly linked to Muc2 depletion have a potential to affect epithelial permeability. Given stated above, *Muc2* knockout model would be particularly helpful to investigate the role of microbiota and metabolism in mitochondrial damage, intestinal barrier disruption and inflammation. It might serve a platform to search for metabolic drugs capable to ameliorate the leaky gut syndrome in the diseases like IBD in clinical remission, IBS, and colon cancer.

## Methods

### Animal housing

The study was conducted in the Center for Genetic Resources of Laboratory Animals at the Federal research center Institute of Cytology and Genetics of The Siberian Branch of the Russian Academy of Sciences (ICG SB RAS), unique identifier of the project RFMEFI62117X0015. All procedures were conducted under Russian legislation according to Good Laboratory Practice standards (directive # 267 from 19.06.2003 of the Ministry of Health of the Russian Federation), inter-institutional bioethical committee guidelines and the European Convention for the protection of vertebrate animals used for experimental and other scientific purpose. All procedures were approved by the Inter-institutional bioethical committee at ICG SB RAS, protocol #18.4 (14.10.2013). All animals had SPF status, which was tested quarterly according to Federation of European laboratory animal science associations (FELASA) recommendations^87^, and the animal colonies tested negative for the pathogens recommended to be checked.

The experiments were performed using C57BL/6JNskrc (our in-house colony of C57BL/6J mice) and *Muc2*^*−/−*^ mouse strains. *Muc2*^*−/−*^ mice were obtained by the rederivation of previously generated *Muc2*^*tm1Avel*^/*Muc2*^*tm1Avel*^ mice^44^ on C57BL/6 genetic background^45^ in SPF CD1 female mice and backcrossing to C57BL/6JNskrc. Mutant mice and their wild-type littermates (*Muc2*^+*/*+^ mice) were obtained by crossing *Muc2*^+*/−*^ females to *Muc2*^+*/−*^ males. *Muc2*^+*/*+^ mice were used in the intestinal permeability assay only, whereas C57Bl/6 (C57BL/6JNskrc) mice served as a control group in all experiments.

Adult 12–14 week-old male mice were housed in groups of the same-sex siblings in individually ventilated cages (Optimice, Animal Care Systems) with birch sawdust as litter and paper cups as shelter. The housing conditions were: 14 h/10 h light/dark photoperiod with 22–24 °C temperature, 30–60% humidity, and 14–16 volumes of air exchange per hour; food (SSniff, Germany) and water were provided ad libitum. SPF *Muc2*^*−/−*^ mice develop signs of colitis before weaning, and some animals tend to develop severe intestinal prolapses, exhibit substantial weight loss and decrease in activity over time. None of the mutant animals used in this study had any of these features.

Animals were euthanized using CO_2_ inhalation. Descending colon samples were taken for histology, cytokine multiplex analysis, real-time PCR, western blot, immunohistochemistry, ATP measurement, OCR and ROS measurement and TEM.

### DNP treatment

Adult 11 week-old male C57Bl/6 mice (N = 8) were provided with 0.8 g/l DNP^88^ in drinking water for 2 days, another group of C57Bl/6 mice (N = 8) was provided regular drinking water. After that, the intestinal permeability assay was performed, and the mice were euthanized with CO_2_ inhalation. Descending colon samples were taken for ATP measurement and immunohistochemistry.

### Histology and clinical scores

Descending colons were fixed in 10% neutral buffered formalin and embedded in paraffin, N = 6 per each mouse strain. Paraffin sections (4 µm) were stained with Periodic acid-Schiff (PAS) stain (BioVitrum, Russia) and with azur-II-eosin. The sections were examined in a blinded manner. Images were taken with an AxioImager.M2 microscope with N-Achroplan 5×/0.15 and 10×/0.25 objectives using an Axiocam 305 color camera (Zeiss, Germany). The number of epithelial cells in the crypts was counted in PAS stained sections. Hyperplasia was defined as the percentage of cells per crypt above the mean number of those counted in the control sctions^49^. Epithelial damage was defined as epithelial cell layer continuity distortion, erosion or ulceration, if present. PMN cells were counted in azur-II-eosin stained sections in 15 fields of view at × 1000 magnification using Plan-Neofluar 100×/1.30 objective to evaluate inflammation severity^89^. The scoring system described by Barthel et al.^89^ and Bergstrom et al.^49^ was used with modifications as follows:

- Hyperplasia (0: < 10%; 1: 10–50%; 2: 51–100%; 3: > 100%),
- Epithelial damage (0: no pathological changes detectable; 1: epithelial desquamation; 2: erosion of the epithelial surface (gaps of 1 to 10 epithelial cells/lesion); 3: epithelial ulceration (gaps of > 10 epithelial cells/lesion),
- PMN cell infiltration (0: < 0.8 cells per field of view, 1: 0.9–3.2 cells per field of view; 2: 3.3–9.6 cells per field of view; 3: > 9.6 cells per field of view).

The maximum score that could result from this scoring system (Total score) was 9.

Mitotic plates were counted on PAS-stained sections at × 1000 magnification at lower, middle and upper thirds of colonic crypts separately per slide (N_C57Bl_ = 3, N_*Muc2−/−*_ = 4).

For clinical score evaluation, eight sex- and age-matched animals per experimental group were weighted, appearance and stool samples were checked manually. Clinical score was applied as described by Van der Sluis et al.^45^ and by Rodrigues et al.^90^ with modifications. Each parameter was scored as follows:

- Weight loss: 0—none, 1—0–17%, 2—18–35%, 3— > 35%.
- Stool: 0—normal droppings, 1 – loose droppings, 2—diarrhea.
- Fecal blood: 0—none, 1—visible blood in rectum, 2—visible blood on fur.
- Appearance: 0—lively/normal, 1—hunched, 2—lethargic, 3—motionless and sickly.

Weight loss of *Muc2*^*−/−*^ mice was calculated taking the sex- and age-matched C57Bl/6 mice as 100%. The maximum score that could result from this scoring system (Total score) was 10.

### Immunology multiplex assay

To measure cytokine levels, a descending colon sample was homogenized in liquid nitrogen (N = 5 for the control, N = 6 for *Muc2*^*−/−*^), 100 μl PBS was added, and then the sample was centrifuged at 12,000 rpm for 15 min at 4 °C. Cytokine concentration in the supernatant was measured using MILLIPLEX MAP Mouse Cytokine/Chemokine Magnetic Bead Panel (Merck, Germany) according to the manufacturer’s recommendations. Detection was performed using Luminex 200 System (Merck, Germany) with xPONENT 3.1. software. Cytokine concentration was normalized to total protein, which was measured as described by Bradford^91^, and is presented as pg of cytokine per mg of total protein.

### Intestinal permeability assay

Intestinal permeability was measured using 4-kDa FITC-Dextran (Sigma-Aldrich, Germany), N = 8 for each group^49^. 100 μl FITC-Dextran (20 mg/ml in distilled water) was administered by oral gavage using a steel feeding tube. After 4 h, 200 μl of blood was collected by orbital sinus puncture. For blood collection, the eyes of the test mice were treated with a drop of an ophthalmic anesthetic (0.5% proparacaine hydrochloride ophthalmic solution, Alcon Laboratories, Alcon-Couvreur N.V. S.A., Belgium). Blood was diluted with PBS containing 0.5% heparin and centrifuged at 3000 rpm for 15 min at 4 °C. 100 µl of supernatant was applied to a 96-well plate, and FITC (485 nm/535 nm) fluorescence was measured using Spark 10 M multimode microplate reader (Tecan Trading AG, Switzerland). Baseline blood plasma fluorescence was determined in mice after oral gavage with water (N = 4 for each genotype) and subtracted from fluorescence obtained after FITC-Dextran gavage. FITC-Dextran concentrations were determined from standard curves generated by serial dilutions of FITC-Dextran.

Adult 10-week-old male C57Bl/6 (N = 4) and *Muc2*^*−/−*^ mice (N = 5) and 18–20-week-old male C57Bl/6 (N = 4) and *Muc2*^*−/−*^ (N = 5) mice were used in order to test age-dependant intestinal permeability using the assay described above. Baseline blood plasma fluorescence after oral gavage with water was determined in 3 mice for each genotype.

Colonic permeability was measured by rectal administration of 4-kDa FITC-Dextran at 2 mg/10 g of body weight^92^. Blood was collected 2 h after FITC-Dextran administration as described above.

### Real-time PCR

To measure the gene expression level, the following procedures were performed as was previously described by Borisova et al.^93^ with some modifications. Total RNA was purified from the descending colon samples using TRIzol reagent (ThermoFisher Scientific, Waltham, MA, USA) according to the manufacturer’s recommendations (N = 6–8 for both lines). Genomic DNA was removed from RNA samples using DNase I (ThermoFisher Scientific, Waltham, MA, USA) according to the manufacturer’s recommendations. RNA concentration was determined with a NanoDrop 2000 spectrophotometer (ThermoFisher Scientific, Waltham, MA, USA). 5–7 µg of RNA was used in the reverse transcription reaction, cDNA synthesis was performed using M-MuLV reverse transcriptase (SibEnzyme, Novosibirsk, Russia) according to the manufacturer’s recommendations. A mix of random hexa-deoxyribonucleotide and Oligo-dT primers were used for reverse transcription. Upon the completion of DNA synthesis, the reaction was diluted with 4 volumes of deionized water. Real-time PCR reaction was prepared using a BioMaster HS-qPCR SYBR Blue (2x) (BioLabMix, Novosibirsk, Russia), 5 µl of cDNA and 250 nM specific primers. Amplification and detection were performed using a CFX96 Touch Real-Time PCR Detection System (BioRad, Hercules, CA, USA). Gene expression was normalized to *Tubb5* (Tubulin, beta 5 class I) mRNA level and calculated as $\Delta {\text{Ct}} = 2^{{({\text{Ct}}_{{Tubb5\;{\text{mRNA}}}} - {\text{Ct}}_{{{\text{gene}}\;{\text{of}}\;{\text{interest}}\;{\text{mRNA}}}} )}}$. Primer sequences used for real-time PCR analyses are shown in Supplementary Table S1.

### Antibodies and immunoblot analyses

For immunoblots, the descending colon samples (N = 6 for both genotypes) were homogenized in RIPA buffer (150 mM NaCl, 1% Nonidet P-40, 0.5% sodium deoxycholate, 0.1% SDS, 25 mM Tris (pH 7.4), 1 mM sodium metabisulphite , 1 mM dithiothreitol, 1 mM phenylmethylsulfonyl fluoride) using plastic pestles. The lysates were centrifuged at 12,000×*g* at 4 °C for 30 min, the supernatants were collected and used to quantify protein concentration as described by Bradford^91^. Total protein extracts were boiled in SDS-PAGE sample buffer, and about 40 μg of total protein was loaded per lane of the 15% acrylamide gel. Rabbit polyclonal anti-Claudin-3 (ab15102, Abcam, Cambridge, UK), rabbit polyclonal anti-Claudin-7 (34-9100, ThermoFisher Scientific, Waltham, MA, USA), rabbit polyclonal anti-β-actin (#PA5-16914, ThermoFisher Scientific, Waltham, MA, USA), and mouse monoclonal anti-GAPDH (MA5-15738, ThermoFisher Scientific, Waltham, MA, USA) antibodies were used at 1:1000. Goat anti-rabbit HRP and goat anti-mouse HRP (#A-11036 and #G-21040 respectively, both ThermoFisher Scientific, Waltham, MA, USA, 1:3500) served as secondary antibodies. Images were captured using an Amersham Imager 600 System (GE Healthcare) and Novex ECL Chemiluminescent Substrate Reagent Kit (ThermoFisher Scientific, Waltham, MA, USA). Immunoblot quantification was performed using ImageJ software.

### Immunohistochemistry

Mice were anaesthetized intraperitoneally (domitor, 0.25 mg/1 kg body weight and zoletil, 15 mg/1 kg body weight), N = 6 per each group. Intracardial perfusion was performed using 15 ml of PBS and 15 ml of 4% PFA per each animal. Descending colon samples were post-fixed overnight in 4% PFA, then kept in 15% sucrose for 24 h and in 30% sucrose for another 24 h. 100 μm sections were prepared using 550 HM Microm cryostat (ThermoFisher Scientific, Waltham, MA, USA). Sections were incubated in 1% bovine serum albumin (BSA) in PBS + 0.15% Triton X-100 + 0.15% Tween-20 for 2 h. The slides were washed three times for 5 min in PBS + 0.1% Triton X-100 (PBST). Primary antibodies were incubated overnight at 4 °C in PBST + 0.1% BSA and washed three times for 5 min each with PBST. Secondary antibodies and Alexa Fluor 568 Phalloidin were incubated for 2 h at room temperature in PBST + 0.1% BSA and washed three times for 5 min each with PBST^94^. The colonic sections were mounted in VectaShield medium with 0.15 µg/ml DAPI. For immunohistochemistry, Alexa Fluor 568 Phalloidin (# A12380, ThermoFisher Scientific, Waltham, MA, USA, 1:1000) was used to detect F-actin, rabbit polyclonal anti-Claudin-3 (ab15102, Abcam, Cambridge, UK, 1:100) were used to detect Claudin-3. Highly cross-absorbed goat anti-rabbit Alexa 488 (#A-11034, ThermoFisher Scientific, Waltham, MA, USA, 1:200) were used as secondary antibodies. Images were obtained using a confocal microscope LSM 710 (Carl Zeiss, Germany) with 20×/0.8 plan-apo and oil immersion 63×/1.40 plan-apo lenses and the ZEN 2012 software. Fluorescence intensity quantification was performed in individual confocal slices using ImageJ software. Claudin 3 staining intensity was measured along the cell membranes, Phalloidin staining was measured within the brush border. At least 30 independent measurements were made for at least three biological replicates per group. Confocal microscopy was performed in the core facility of the Institute of Molecular and Cellular Biology SB RAS.

### TEM

Descending colon samples (N = 2 for both groups) were fixed in 2.5% glutaraldehyde solution in 0.1 M sodium cacodylate buffer (pH 7.4) for 1 h at room temperature, washed and postfixed in 1% osmium tetroxide with 0.8% potassium ferrocyanide for 1 h. Fixed samples were contrasted with 1% uranyl acetate in water, dehydrated and embedded in epoxy resin (Epon 812). Semi-thin cross sections were prepared, stained with methylene blue and analyzed with an Axioscope-4 microscope (Zeiss, Germany). Ultrathin section (60 nm) for transmission electron microscopy were obtained with a diamond knife on Leica EM UC7 ultramicrotome (Leica, Austria) and then examined with a JEM1400 transmission electron microscope (JEOL, Japan)^95^. TEM was performed at the Interinstitutional Shared Center for Microscopic Analysis of Biological Objects (ICG SB RAS, Novosibirsk).

Measurements were taken in epithelial cells from the middle part of colonic crypts. For statistical analysis, the morphological structures were measured using iTEM software (OlympusSIS, Germany) in randomly chosen sections (from 30 to 100 independent measurements per genotype for each structure). TJs and AJs were considered defective if swelling or “bubbling” were clearly visible. For TJ width assessment, three independent measurements were made per one TJ. For AJ width assessment, up to 10 independent measurements were made per one AJ. Desmosomes were counted per one lateral membrane per cell and were considered defective if they had a ‘semi-desmosome’ appearance. Intercellular space was measured as a distance between the membranes of the two adjacent cells at 100 randomly chosen regions for each test group. Rootlet/microvillus ratio was calculated as a length of the rootlet (part of a filamentous actin bundle located within the cell body)/length of the microvillus. Mitochondria were counted for 30 cells per each genotype, cristae density was calculated per 0.25 µm^2^ of mitochondrial matrix. Mitochondria were considered empty, if less than 3 cristae were visible within mitochondrial matrix.

### ATP measurement

ATP content in distal colon samples of C57Bl (N = 15 and 8), *Muc2*^*−/−*^ (N = 15), and C57Bl/6 + DNP (N = 8) mice was determined as described previously with minor modifications^96^. Briefly, tissue samples were placed in 200 µl 70% ethanol (v/v) containing 2 mM EDTA (pH 10.9) and snap-frozen in liquid nitrogen. The samples were thawed and homogenized by sonication, ATP extracts were diluted 10 times with a buffer containing 0.1 M Tris–HCl and 2 mM EDTA (pH 7.8). ATP was measured using a commercially available ATP Assay Kit (#119107, Merck, Germany) as per manufacturer’s instructions with luminometer Berthold TriStar LB 941 (Berthold Technologies, Germany). ATP concentration was normalized to total protein, which was measured as described by Bradford^91^ and presented as fold change over control.

### Colonic crypt isolation

Crypts were isolated as described previously^97^ with modifications. Briefly, colonic samples from 3 C57Bl/6 and 3 *Muc2*^−/−^ males were removed and placed in a Petri dish with ice-cold PBS. Feces were flushed with ice-cold PBS using a 5-ml syringe, peritoneal fat was removed with scissors. The colon was dissected longitudinally and washed three times with cold PBS. Then, it was cut in 5 mm pieces, placed in a 50 ml tube and washed three times with 30 ml of ice-cold PBS. After the last wash, tissue samples were transferred to the pre-warmed 3 mM EDTA/1 mM DTT/PBS buffer and incubated at 37 °C in a water bath for 30 min. The released crypts were filtered through a 70 µm strainer, briefly washed with PBS and spun at 500 g for 5 min. The crypts were resuspended in 1 ml of pre-warmed DMEM containing 10% FBS and 1% penicillin–streptomycin (all ThermoFisher Scientific, Waltham, MA, USA) and used for further analyses.

### OCR analysis

Eight-well Seahorse XFp cell culture microplates (#101037-004, Agilent Technologies, Santa Clara, CA, USA) were pre-coated with 10 μl of Matrigel (#354277, Corning, New York, USA), diluted 1:40 with DMEM, polymerized at 5% CO2, 37 °C and kept at 4 °C for 24 h. On the day of analysis, Matrigel-precoated plates were pre-warmed at 5% CO2, 37 °C and used to seed 50 µl of isolated colonic crypts. An aliquot of each crypt sample was used for protein assay. The plates were used for OCR measurement with Seahorse XF analyzer (Agilent Technologies, Santa Clara, CA, USA). The program was as follows: Calibrate; Equilibrate; Loop start: 3× Mix: 3 min, Measure: 3 min, Loop end; Inject: port A (DNP, 100 μM), Loop start: 3× Mix: 3 min, Measure: 3 min, Loop end; End. OCR was normalized on total protein measured with Bradford assay^91^ in a corresponding crypt sample aliquot. The first baseline measurement was used to compare OCR between genotypes. The entire dataset was used for repeated measures analysis within each genotype to evaluate the effect of DNP indicative of the spare respiratory capacity.

### ROS measurement

Isolates crypts (200 µl) were treated with 0.5 µl of CellROX Deep Red reagent (#C10422, ThermoFisher Scientific, Waltham, MA, USA) in DMEM for 30 min at 5% CO2, 37 °C. Crypts were then fixed with 4% buffered formaldehyde solution for 10 min at room temperature, washed 2 times with PBS and stained with DAPI (#D1306, ThermoFisher Scientific, Waltham, MA, USA) in PBS for 5 min at room temperature. ROS were identified by measuring fluorescence intensity with a Spark 10 M microplate reader and normalized to the DAPI fluorescence.

### Statistics

The data were tested for normality using the Kolmogorov–Smirnov test. All data are presented as mean ± standard error of the mean (SEM). Normally distributed data were analyzed using Student’s *t*-test for independent samples. Not normally distributed data were analyzed using Mann–Whitney *u* test. Percentage of TJs, AJs, and desmosomes with defects and the percentage of intercellular spaces wider than 25 nm were analyzed using the χ^2^ test. The effect of DNP on OCR was analyzed using Friedman test.

## Supplementary information

Supplementary Information.
