# Supplementary material for: Mucin-2 knockout is a model of intercellular junction defects, mitochondrial damage and ATP depletion in the intestinal epithelium
Source: Sci Rep. 2020 Dec 3;10:21135. doi: 10.1038/s41598-020-78141-4 (PMC7713437; doi:10.1038/s41598-020-78141-4)
Supplement: Supplementary file 1 — Supplementary Information. [file 41598_2020_78141_MOESM1_ESM.pdf]

Supplementary Information

Mucin-2 knockout is a model of intercellular junction defects, mitochondrial damage and ATP depletion in the intestinal epithelium.

Mariya A. Borisova[1,#], Kseniya M. Achasova[2,3,4,#], Ksenia N. Morozova[1], Evgeniya N. Andreyeva[2], Ekaterina A. Litvinova[3,4], Anna A. Ogienko[2], Maryana V. Morozova[2,3,4], Mariya B. Berkaeva[2] , Elena Kiseleva[1], Elena N. Kozhevnikova[2,3,4,*]

[1] The Federal Research Center Institute of Cytology and Genetics of The Siberian Branch of the Russian Academy of Sciences, Novosibirsk 630090, Russian Federation;

[2] Institute of Molecular and Cellular Biology, The Siberian Branch of the Russian Academy of Sciences, Novosibirsk, 630090, Russian Federation;

[3] Scientific Research Institute of Physiology and Basic Medicine, Novosibirsk, 630117, Russian Federation;

[4] Siberian Federal Scientific Centre of Agro-BioTechnologies of the Russian Academy of Sciences, Krasnoobsk, Novosibirsk region, 630501, Russian Federation;

[#] Equal contribution

***** Correspondence: kozhevnikovaen@physiol.ru; Tel.: (+7-383-373-01-82)

Supplementary figure S1. Colon epithelium proliferation, intestinal permeability, and gene expression in *Muc2* knockout mice. A. Mitotic plates in the lower, middle and upper thirds of the colon crypth. * = $p < 0.05$, Mann-Whitney *u* test. B. Intestinal permeablity in $Muc2^{-/-}$ wild-type littermates ($Muc2^{+/+}$). Intestinal permeability assay in 10-week old and in 18-20-week old mice, and colonic permeablity upon rectal administration of FD-4. * = $p < 0.05$, Mann-Whitney *u* test. C. Apical junction complex proteins, *matrix metallopeptidases 2 and 9, mitochondrially encoded NADH dehydrogenase subunits 2* and *6* and *leukotriene C4 synthase* gene expression (normalized to β-tubulin (*Tubb5* gene), fold change.

Supplementary figure S2. Examples of structural defects in TJs upon *Muc2* knockout as compared to control.

Supplementary figure S3. *Muc2* knockout results in the increased number of defective desmosomes and expanded intercellular space. A. TEM of desmosomes in the descending colon epithelium of C57Bl/6 and $Muc2^{-/-}$ mice. B. TEM of intercellular spaces in the descending colon epithelium of C57Bl/6 and $Muc2^{-/-}$ mice. C. Morphometric analysis: desmosomes per lateral cell membrane (*** = $p < 0.001$, Student's t-test), the percentage of the defective desmosomes (*** = $p < 0.001$, $\chi2$ test), intercellular space width (*** = $p < 0.001$, Student's t-test), intercellular spaces wide than 25 nm, percentage (*** = $p < 0.001$, $\chi2$ test). Dm – desmosome, Id – intercellular space.

Supplementary figure S4. Original Western blot images used in this study.

Supplementary table S1.Primers used in the study.

Target	Primer name	Primer sequence 5' -> 3'
Mouse *β-tubulin (Tubb5)*	betaTub F	TGAAGCCACAGGTGGCAAGTAT
	betaTub R	CCAGACTGACCGAAAACGAAGT
Zo-1	ZO-1 F	TCTCCACATACATTCCAAGGGC
	ZO-1 R	GAGTTGGTGGTCTGAAAGTTGC
E-cadherin	Cdh1 F	CCAAGCACGTATCAGGGTCA
	Cdh1 R	TGTATTGCTGCTTGGCCTCA
occludin	Ocln F	TTATCTTGGGAGCCTGGACATT
	Ocln R	CTGGCTGAGAGAGCATCGG
claudin 1	Cldn1 F	ATTTCAGGTCTGGCGACATTAGT
	Cldn1 R	AGAGGTTGTTTTCCGGGGAC
claudin 2	Cldn2 F	CCTCATCAAAACTTCAGCACCG
	Cldn2 R	AAATGGCTTCCAGGTCAGCATA
claudin 3	Cldn-3 F	TTTCTATAACCCGTTGGTGCCC
	Cldn-3 R	ATCTTGGTGGGTGCATACTTGT
claudin 4	Cldn4 F	CCCACCCACCTACCCTACTAAT
	Cldn4 R	TTAGCAAGACAGTGCGGAAAAG
claudin 7	Cldn7 F	CCCTCCACTTCTTTGGGTAGTC
	Cldn7 R	GAAGCGACACTCTCACAGCA
claudin 8	Cldn8 F	GGCCTGGGGATAAAAGAGAAGG
	Cldn8 R	CAAAGCAGGATAGCAGAAAGCC
claudin 12	Cldn12 F	CGCTCCCGTTTCCGAGTCTT
	Cldn12 R	CTCTGATCCCCTTTCCCTGTGC
claudin 15	Cldn15 F	CGTGGGCAACATGGATCTCT
	Cldn15 R	CCAGTTCATACTTGGTTCCAGC
matrix metallopeptidase 2	MMP2 F	TTCTATGGCTGCCCCAAGGA
	MMP2 R	GGTGTGTAACCAATGATCCTGTATG
matrix metallopeptidase 9	MMP9 F	AAAGGCAGCGTTAGCCAGAA
	MMP9 R	ACAAGTATGCCTCTGCCAGC
mitochondrially encoded NADH dehydrogenase 2	ND2 F	AGGGGCATGAGGAGGACTTA
	ND2 R	TTGAGTAGAGTGAGGGATGGGT
mitochondrially encoded NADH dehydrogenase 6	ND6 F	CAACGCCTGAGCCCTACTAA
	ND6 R	AGGACTGGAATGCTGGTTGG
leukotriene C4 synthase	Ltc4 F	TCTTCCGAGCCCAGGTAAACT
	Ltc4 R	GACTAGCAAGCCCAGTGCAG

A

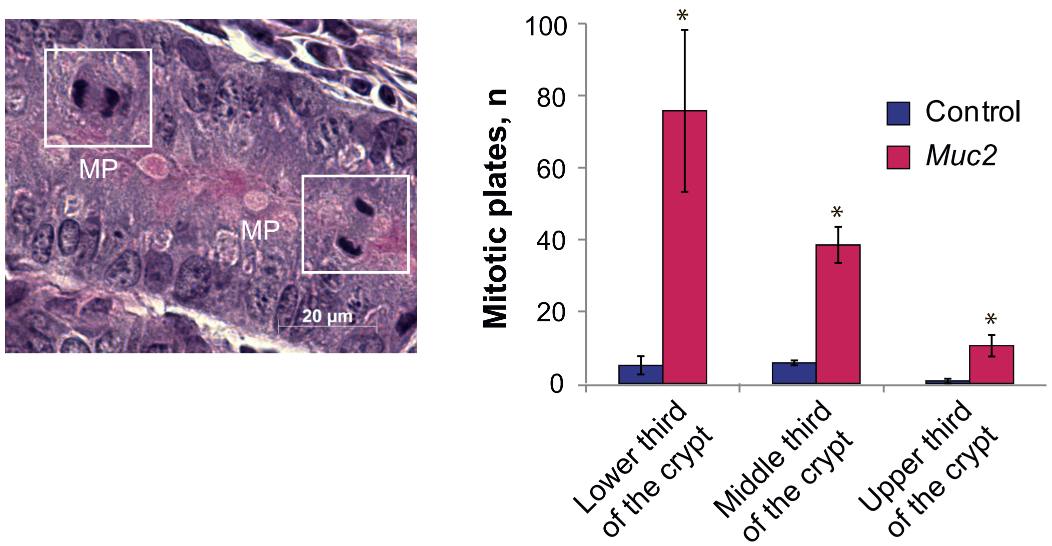
MP
MP
20 µm
Mitotic plates, n
100
80
60
40
20
0
*
*
*
Control
Muc2
Lower third of the crypt
Middle third of the crypt
Upper third of the crypt

B

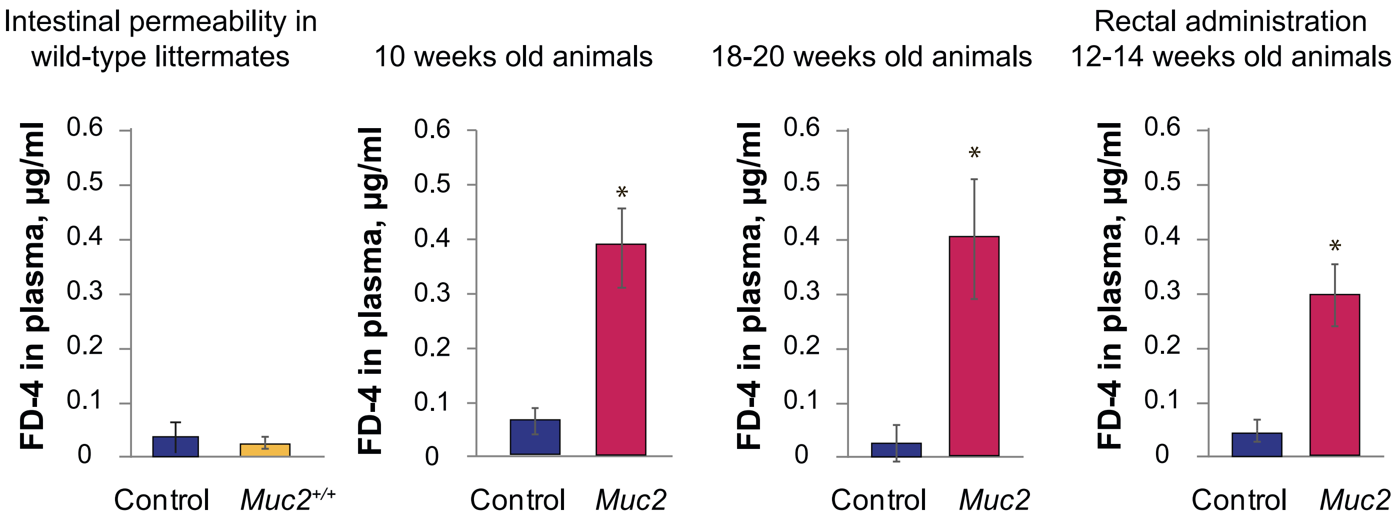
Intestinal permeability in wild-type littermates
10 weeks old animals
18-20 weeks old animals
Rectal administration 12-14 weeks old animals
FD-4 in plasma, µg/ml
0.6
0.5
0.4
0.3
0.2
0.1
0
*
Control
Muc2+/+
Muc2

C

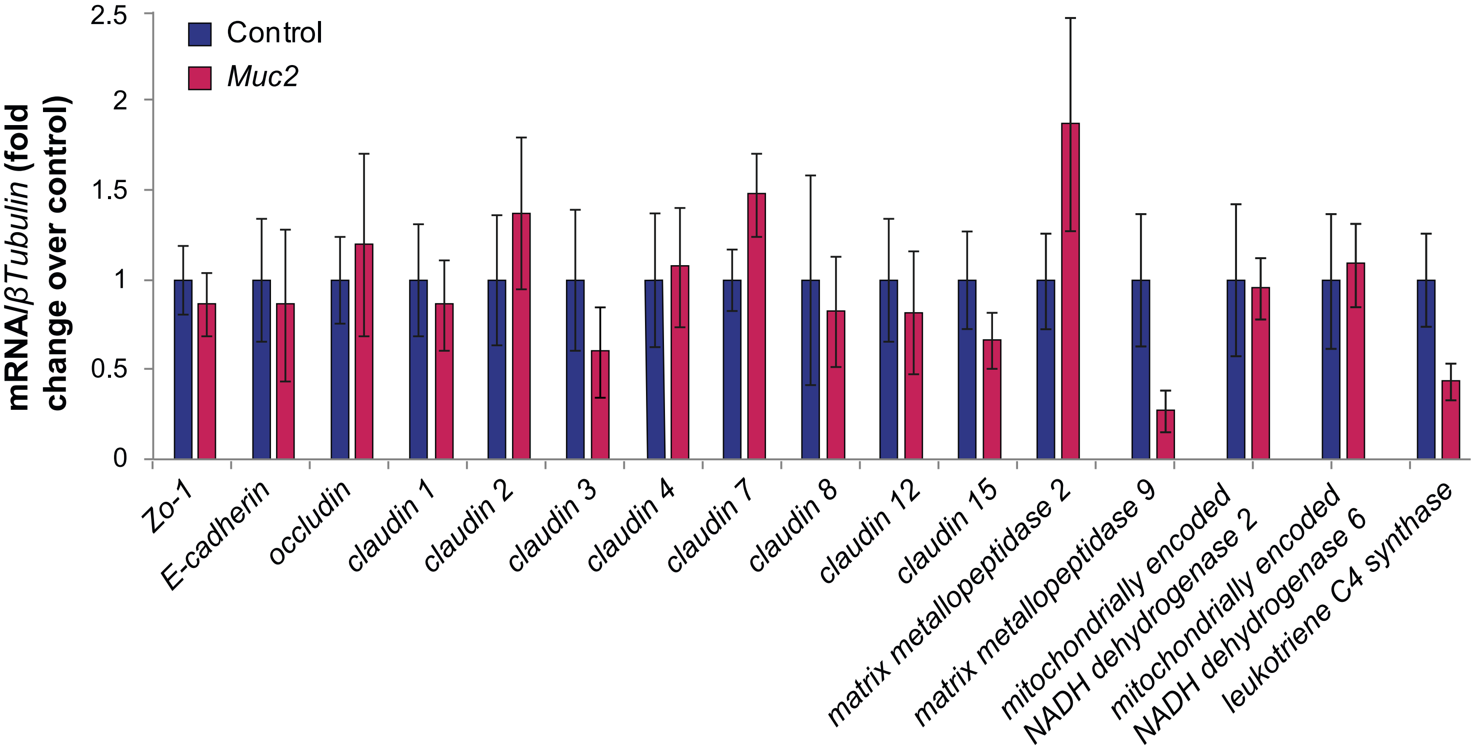
mRNA/βTubulin (fold change over control)
2.5
2
1.5
1
0.5
0
Control
Muc2
Zo-1
E-cadherin
occludin
claudin 1
claudin 2
claudin 3
claudin 4
claudin 7
claudin 8
claudin 12
claudin 15
matrix metallopeptidase 2
matrix metallopeptidase 9
mitochondrially encoded NADH dehydrogenase 2
mitochondrially encoded NADH dehydrogenase 6
leukotriene C4 synthase

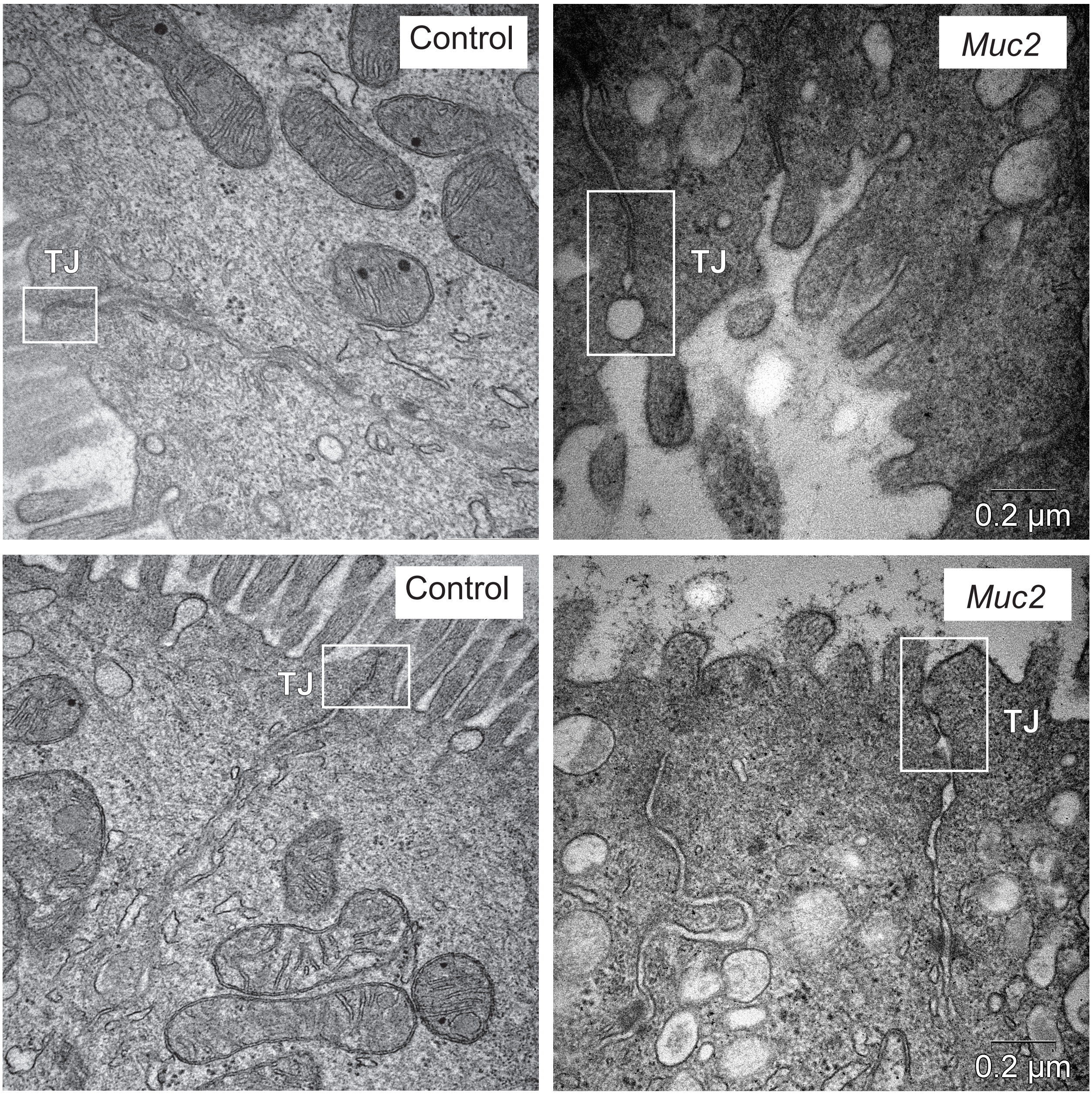

Control
TJ
Muc2
TJ
0.2 μm
Control
TJ
Muc2
TJ
0.2 μm

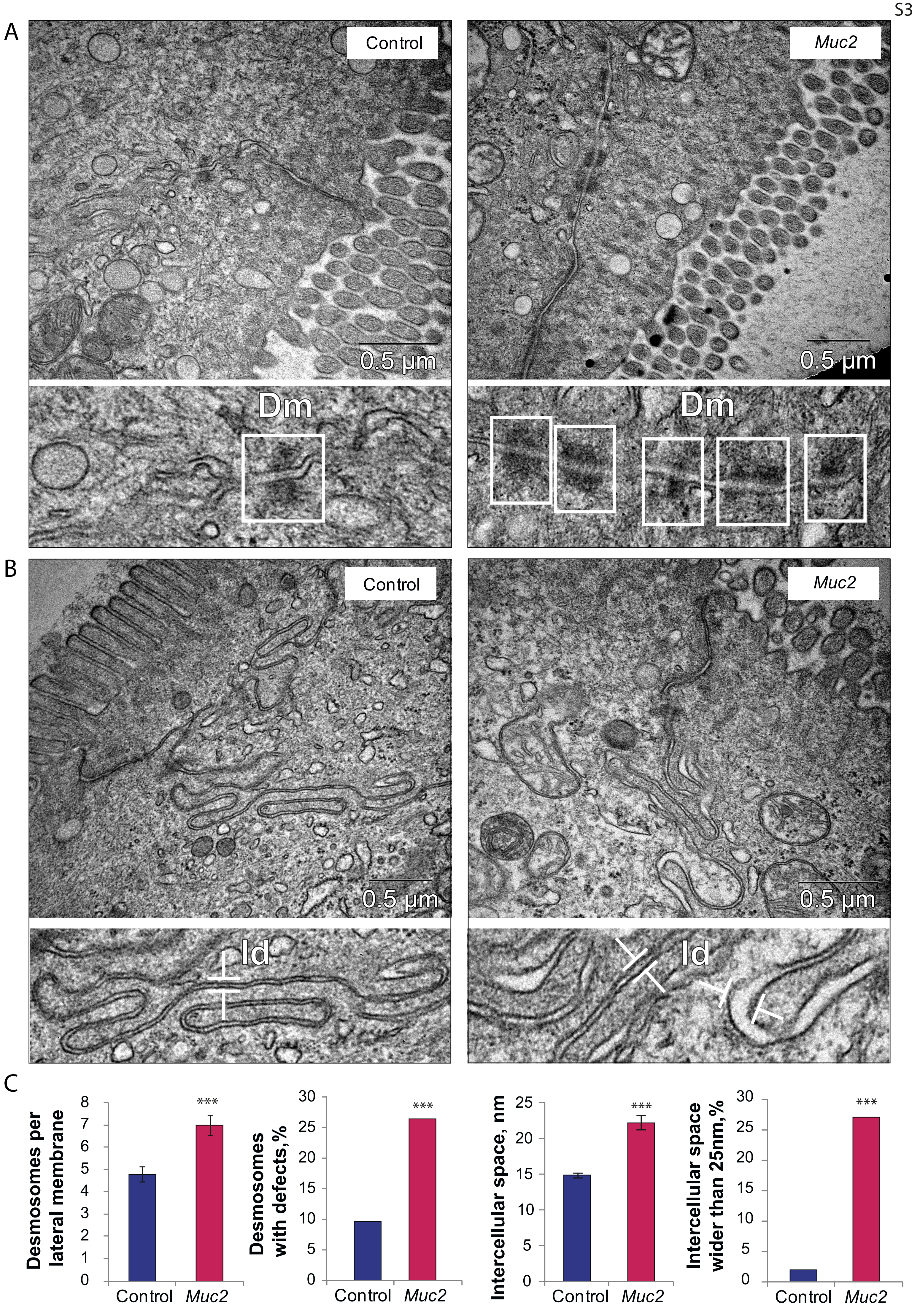
A
Control
Muc2
0.5 µm
0.5 µm
Dm
Dm
B
Control
Muc2
0.5 µm
0.5 µm
Id
Id
C
Desmosomes per lateral membrane
0
1
2
3
4
5
6
7
8
***
Control
Muc2
Desmosomes with defects,%
0
5
10
15
20
25
30
***
Control
Muc2
Intercellular space, nm
0
5
10
15
20
25
***
Control
Muc2
Intercellular space wider than 25nm,%
0
5
10
15
20
25
30
***
Control
Muc2

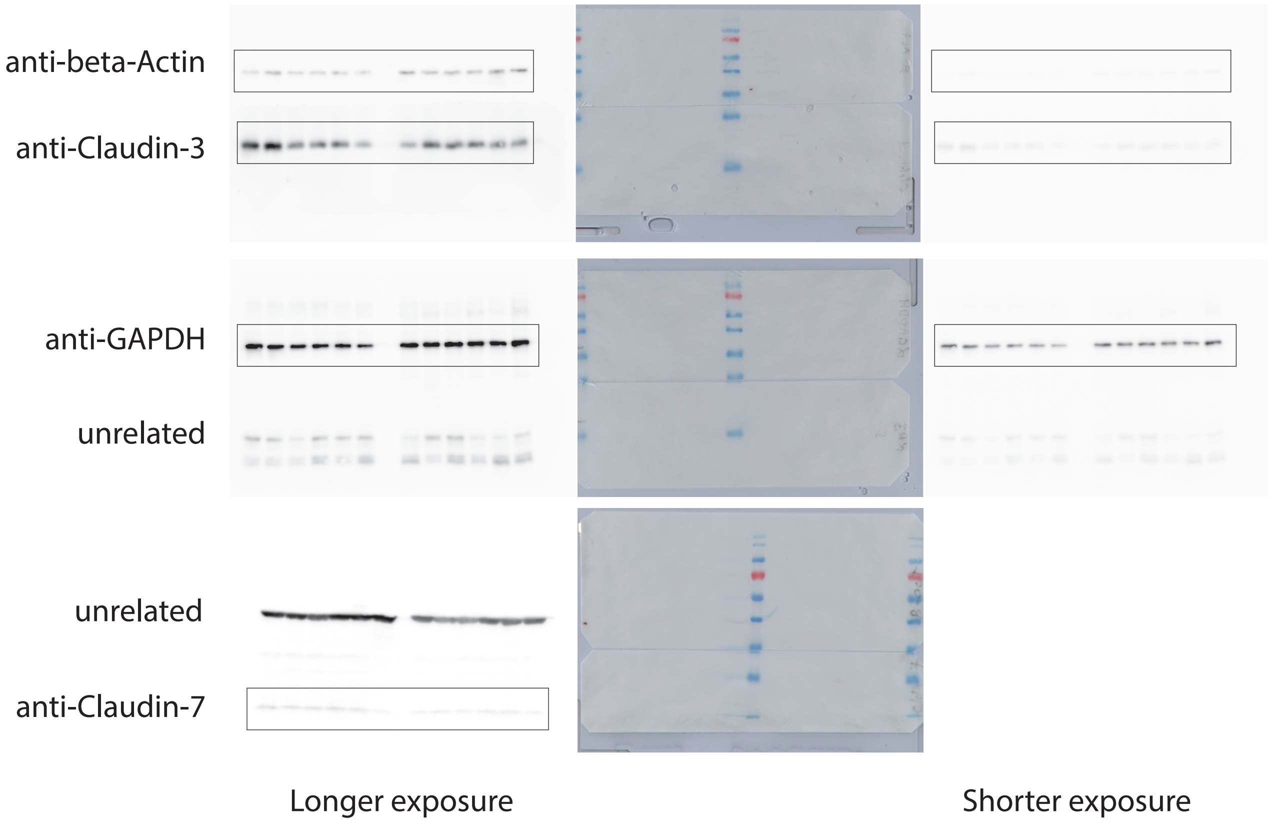
anti-beta-Actin
anti-Claudin-3
anti-GAPDH
unrelated
unrelated
anti-Claudin-7
Longer exposure
Shorter exposure
